# Promising Therapeutics with Natural Bioactive Compounds for Improving Learning and Memory — A Review of Randomized Trials

**DOI:** 10.3390/molecules170910503

**Published:** 2012-09-03

**Authors:** Hemant Kumar, Sandeep Vasant More, Sang-Don Han, Jin-Yong Choi, Dong-Kug Choi

**Affiliations:** 1 Department of Biotechnology, Konkuk University, Chungju 380-701, Korea; 2 Department of Neurology, School of Medicine, Konkuk University, Chungju 380-704, Korea

**Keywords:** learning and memory, cognition, clinical trials, herbal medicines, panax gingseng, EGb 761, BT-11, GK501, asarone, tofu

## Abstract

Cognitive disorders can be associated with brain trauma, neurodegenerative disease or as a part of physiological aging. Aging in humans is generally associated with deterioration of cognitive performance and, in particular, learning and memory. Different therapeutic approaches are available to treat cognitive impairment during physiological aging and neurodegenerative or psychiatric disorders. Traditional herbal medicine and numerous plants, either directly as supplements or indirectly in the form of food, improve brain functions including memory and attention. More than a hundred herbal medicinal plants have been traditionally used for learning and memory improvement, but only a few have been tested in randomized clinical trials. Here, we will enumerate those medicinal plants that show positive effects on various cognitive functions in learning and memory clinical trials. Moreover, besides natural products that show promising effects in clinical trials, we briefly discuss medicinal plants that have promising experimental data or initial clinical data and might have potential to reach a clinical trial in the near future.

## 1. Introduction

Learning is the experience-dependent acquisition of knowledge and skills, whereas memory is the retention and retrieval of facts or events composed of experiences. Memory disorders can range from mild to severe and can be progressive (neurodegenerative disease) or immediate (brain injury). Almost all are linked with some damage to neuroanatomical structures, either in part or full, which hinders acquisition (learning), consolidation (storage of labile stable memory), and retrieval (recall). Alzheimer’s disease (AD) is a complex, multifactoral, progressive, neurodegenerative disease and represents the largest group of people with learning and memory impairments. AD primarily affects the elderly population and accounts for 50–60% of dementia cases in persons >65 years of age. Physiological senescence is normally associated with a decline in cognition and motor skills, thereby decreasing the quality of life in elderly people. Forebrain and hippocampal atrophy [1,2], decreases in acetylcholine levels [3], cholinergic hypofunction [4,5], preferential age-dependent degeneration of basal forebrain cholinergic neurons, and attenuation of neurotrophic signaling in sensory neurons [5] are some of the important factors that might contribute to learning and memory impairment in the elderly. 

Medicine systems such as Ayurveda and Traditional Chinese Medicine (TCM) are based on the use of herbal compounds to treat aging and memory loss. More than 150 plants have been used to improve learning and memory, based on the direct or indirect traditional belief of various preparations and mixtures [6]. Synthetic drugs are known to cause undesirable adverse effects [7,8], whereas natural products are considered safe and effective. Herbal medicines are becoming popular for improving quality of life with either no or limited side effects. The influence of natural products is quite marked in drug discovery; of 1,335 approved drugs from the 1940s to date, 59 (4%) are derived from natural products, and 299 (22%) are derived from a natural product (semisynthetic modification) [9]. 

In this review, we will discuss available clinical data of herbal compounds for disorders involving learning and memory impairment. We have chosen those natural products for which substantial clinical data are available for improving cognition. Besides natural products in clinical trials, we have listed those plants whose experimental data or initial clinical data seem promising and have potential to reach a clinical trial in the near future.

## 2. Bioactive Compounds That Improve Learning and Memory

### 2.1. Galantamine from Galantus, Narcissus, and Leucojum spp.

**Figure 1:**
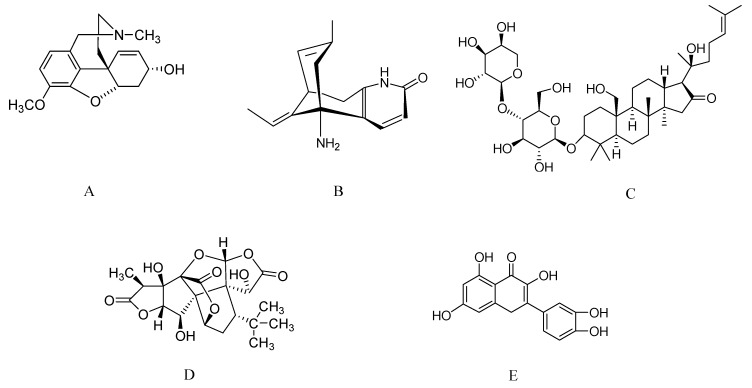
Molecular structure of galantamine (**A**), huperzine A (**B**), bacoside A (**C**), ginkgolide B (**D**), and quercetin (**E**).

Galantamine (galanthamine, Figure 1A) is an alkaloid derivative isolated from several members of the Amaryllidaceae family such as *Galanthus* spp. (snowdrop) *Narcissus* spp. and *Leucojum* spp. It is an approved anticholinesterase drug for patients with AD. Galantamine modulates nicotinic receptors [10,11] and potentiates nicotinic neurotransmission in addition to inhibiting acetylcholinesterase (AChE) [12]. Galantamine binds to an allosteric binding site on nicotinic receptors, thereby potentiating the response of these receptors to the natural agonist, acetylcholine [10]. In initial double blind, placebo controlled trials performed across several countries in patients with mild to moderate AD, the 11-item AD Assessment Scale-Cognitive subscale (ADAS-Cog 11), Clinician’s Interview-Based Impression of Change plus Caregiver Input (CIBIC-plus) were used as assessments to test cognitive function. Galantamine was administered in a dose range of 8–32 mg/kg initially, with maintenance doses of 24 or 32 mg/kg, and showed benefits on cognitive function and improved basic activities of normal living than those of a placebo after 3 [13], 5 [14], and 6 months [15,16] (Table 1). These studies show that slow dose escalation enhances the tolerability of galantamine and minimizes the incidence and severity of adverse events. In another study, patients were randomly assigned to galantamine 24 mg/day (n = 396) or placebo (n = 196) groups to determine the efficacy of galantamine in patients with vascular dementia (VaD) or AD combined with cerebrovascular disease. Galantamine showed greater efficacy than placebo on the ADAS-Cog 11 and CIBIC-plus. Activities of daily living (ADL) and behavioral symptoms were also significantly improved compared to those in the placebo group. Galantamine was well tolerated and showed a therapeutic effect in all key areas of cognitive and non-cognitive abilities in patients with dementia [17]. From this study, it is clear that galantamine has efficacy in patients with mild to moderate AD and VaD or AD combined with cerebrovascular disease. Another study was undertaken to determine the efficacy of galantamine in patients with severe AD. Patients (n = 207) with severe AD (Mini-Mental State Examination [MMSE] scores of 5–12 points), aged 84 ± 6 years were randomly assigned to receive galantamine, titrated initially at 24 mg/day, or placebo (n = 200). Co-primary efficacy measures to assess cognitive function and the ability to undertake normal ADLs were the Severe Impairment Battery (SIB) and the Seven-item Minimum Data Set-Activities of Daily Living (MDS-ADL), respectively. Mean SIB scores increased (improved) by 1.9 points with galantamine administration and decreased (worsened) by 3.0 points with placebo. Mean MDS-ADL self-performance score decreased by 1.2 points and 1.6 points, respectively. Overall galantamine improved cognitive function but failed to significantly improve the co-primary parameter of overall ADL [18]. A minimum change of 4-points on the ADAS-Cog 11 at 6 months is considered clinically important, but this cut-off point has been little studied in relation to clinical meaningfulness. Therefore, to investigate the extent to which a 4-point change classifies goal attainment by individual patients, a 4-month, multi-center, parallel-group, double-blind, placebo-controlled study was undertaken in patients with mild-moderate AD. Secondary analysis of the video imaging synthesis of treating AD was also used in this study. ADAS-Cog 11 responses at 6 months were compared with outcomes on the CIBIC-plus, the Patient/Career-Goal Attainment Scale (PGAS), and Clinician-Goal Attainment Scale (CGAS) for administered galantamine to 130 patients with mild-moderate AD (4-month open-label follow-up). Thirty-seven of the 99 patients improved by 4-points on the ADAS-Cog 11 at 6 months, and 16/99 showed a 4-point worsening in the galantamine group. ADAS-Cog 11 scores correlated modestly with changes on the CGAS, the PGAS, and the CIBIC-plus. The results of this study revealed that substantial individual misclassification between the ADAS-Cog 11 and clinical measures suggests no inherent meaning to a 4-point ADAS-Cog 11 change for any given patient [19].

Furthermore, to evaluate the effects of galantamine withdrawal, and compare this with uninterrupted therapy, two 6-week double-blind withdrawal studies (studies 1 and 2) were performed in patients with mild to moderate AD. In study 1 (GAL-USA-11; n = 723), patients continuously treated with 16 mg/day galantamine exhibited improved ADAS-Cog 11 scores compared to those of a parent trial baseline. Over the same period, patients switched from galantamine to placebo and those who had received continuous placebo exhibited deteriorations of 0.7 and 1.2 points, respectively.

**Table 1:** Natural bioactive compounds in clinical trials.

Bioactive Compound/Intervention	Study design	Memory test	Result/Activity	Ref.
Galantamine	Patients with Mild to moderate AD, R, DB, PC (3 months/n = 386)	CIBIC-plus and ADAS-cog	Improved cognitive function and basic activities of normal living than placebo in AD patients.	[13]
Galantamine (8, 16 & 24 mg/day)	Patients with Mild to moderate AD, R, DB, PC, M (5-month)	ADAS-cog and CIBIC-plus	Benefits the cognitive, functional, and behavioral symptoms of AD as compared with placebo.	[14]
Galantamine (24 or 32 mg/day)	Patients with Mild to moderate AD, M, DB, (6-month/n = 636)	ADAS-cog and CIBIC-plus	Improved cognition and global function, better outcome on CIBIC-plus and ADAS-cog.	[15]
Galantamine (24 or 32 mg)	Patients with Mild to moderate AD, R, DB, PC blind, parallel group, trial (6-month/n = 653)	ADAS-cog	Better scores on the disability assessment for dementia, slowed the decline of functional ability as well as cognition in subjects with mild to moderate AD as compared with placebo.	[16]
Galantamine (24 mg/day)	Patients with Vascular dementia, M, DB, (n = 592)	ADAS-cog and CIBIC-plus	Therapeutic effect on all key areas of cognitive and non-cognitive abilities with improved activities of daily living and behavioral symptoms were also significantly improved in dementia patients.	[17]
Galantamine (24 mg/day)	Patient with severe AD, R, DB, PC, blind (n = 207)	SIB and MDS-ADL	Improvement in memory, praxis, and visuospatial ability.	[18]
Huperzine A (0.2 mg twice daily)	R, DB, PC with AD (n = 28)	MMSE, ADL, HDS and WMI	Improved memory test scores over the individuals receiving the placebo.	[20]
Huperzine A (0.4 mg daily)	Subjects with benign vascular dementia, and AD (n = 80)	MQ test	Significant improvement in MQ test as compared to control group.	[21]
Huperzine Alpha (400 μg/day)	Subjects with diagnosis of possible or probable AD (n = 100/12 weeks)	ADL &ADAS-Cog	Remarkably improves the cognition, behavior, ADL, and mood of AD patients as assessed by ADAS-Cog.	[22]
Huperzine A (200 & 400 μg; twice daily)	Patients with mild to moderate AD, M, R (16 weeks/n = 210)	ADAS-Cog	Huperzine A 400 μg and not at 200 μg has cognitive effect in patients with mild to moderate AD.	[23]
Huperzine A (0.1 mg twice daily)	Patients with mild to moderate VaD, R, DB ,PC (12 weeks/n = 78)	CDR, MMSE, and ADL	Huperzine A showed significant improvement in cognitive functions of all test.	[24]
Bacognize^®^ (300mg; twice daily)	Patients with AD, R, DB, PC and M (6 months)	MMSE	Improvement in attention, language, reading, writing & comprehension.	[25]
*Bacopa monniera*	Healthy individuals, DB, PC (n = 46)	Well-validated neuropsychological tests	Significant improvement in information processing and memory consolidation, & in state anxiety.	[26]
*Bacopa monniera* (2 × 150 mg)	Healthy individuals, DB, PC (90 days/n = 127)	Neuropsychological testing using the Cognitive Drug Research cognitive assessment system	Improves partial working memory and reduced number of false positives in the rapid visual information processing task.	[27]
*Bacopa monniera* (300 mg)	65 or older year individuals, R, DB, PC (12weeks/n = 54)	AVLT, DAT, and WAIS	Enhances AVLT delayed word recall memory scores and also improves ability to ignore irrelevant information as assessed by Stroop test.	[28]
BT-11 (extracted from roots of *Polygala tenuifolia*)	Healthy elderly individuals, R, DB, PC (n = 28)	CERAD and MMSE	Treatment by BT-11, increased CERAD scores, word list recognition, constructional recall and praxis, and modified Boston naming test.	[29]
BT-11	Healthy individuals, R, DB, PC (4 weeks)	K-CVLTSOPT	Improvement in verbal memory and working memory.	[30]
EGb 761 (240 mg)	Patients with AD and multi-infarct dementia, R, DB, PC (24 weeks/n = 216)	CGI and NAB	Effective in Alzheimer and multi-infarct dementia.	[31]
EGb 761 (240 mg/day or 160 mg/day)	Patient with AD or VaD or AAMI, R, DB, PC (n = 214/24 weeks)	SKT, CGI and NAI-NAA	EGb 761 is not beneficial for dementia patients.	[32]
EGb 761 (*Ginkgo* extract - 180 mg/day)	Aged subjects with no history of significant neurocognitive dysfunction (6 weeks)	Stroop Color and Word Test color-naming task	Significantly showed improvement on a task assessing speed of processing abilities.	[33]
Standardized extract of *Ginkgo biloba* (GK501) & Standardized extract of *Panax ginseng* (G115) (60 mg - Capsule)	Healthy individuals, DB, PC (14 week/n = 256)	Tests for attention and memory from the Cognitive Drug Research computerized cognitive assessment system	Significantly to improve an Index of Memory Quality, memory, including long-term and working memory.	[34]
Capsulated aqueous extract of *C. asiatica* (250, 500, and 750 mg)	Healthy individuals, R, DB, PC (2 months/n = 28)	Computer assisted technique	Cognitive enhancing effect observed.	[35]
Ginseng (400 mg)	Healthy young volunteer, R, DB, PC, balanced, cross-over (n = 20)	CDR two serial subtraction mental arithmetic tasks	Improvement in the speed and accuracy of memory and attentional tasks.	[36]
Panax ginseng extract (G115) (400 mg)	Healthy middle aged individuals, DB, PC balanced trail (n = 30)	Cognitive and mood performance test	Improvement speed of attention and tasks associated with episodic memory performance.	[37]
Cereboost(*P. quinquefolius* standardized to 10.65% ginsenosides)	Healthy young volunteer, R, DB, PC, crossover trial (n = 32)	Parameters for mood and neurocognitive effect	Improvement working memory performance, reaction time accuracy and calmness.	[38]
G115 (200 mg & 400 mg)	Healthy young volunteer, R, DB, PC, crossover trail (n = 30)	COGNITIVE BATTERY (Bond-Lader visual analogue scales, Computerised Corsi block tapping task, N-back task and Random number generation task	Modulation of cognitive function and mood.	[39]
HT100 (proprietary North American ginseng extract)	Individuals with schizophrenia, DB, PC (4 weeks/n = 64)	Letter-Number Span Test and Visual Pattern Test	Significant improvement in visual working memory.	[40]
LGNC-07 (combination of green tea extract and L-Theanine/1,680 mg)	MCI subjects, DB, PC (16 weeks/n = 91)	Rey–Kim memory test and Stroop color-word test	Significant improvement in selective attention, cognitive alertness, memory and verbal reading.	[41]
Green Tea	Cross-sectional trial (n = 1003)	MMSE	Higher consumption of green tea lowers prevalence of cognitive impairment.	[42]
Total Tea	Cross-sectional and longitudinal (n = 2501)	MMSE	Total tea consumption lowers risk of cognitive impairment and cognitive decline.	[43]
Total Tea	Cross-sectional trial (n = 716)	MMSE	Total tea consumption improves global cognition, memory, executive function, and information processing speed.	[44]
*Salvia officinalis* extract (60 drops/day)	Patients with mild to moderate AD, DB, R, PC (n = 424 months)	ADAS-cog & CDR	Improvement in cognitive functions.	[45]
Extract of *Salvia officinalis* (167, 333, 666 and 1332 mg)	Patients with mild to moderate AD, DB, R, PC (n = 20)	CDR	Enhancement of secondary memory performance.	[46]
*S. officinalis* aroma	Healthy individuals, SB, one factor, independent group trial (n = 135)	CDR & Bond-Lader mood scale	Significantly increases the alertness in mood and quality of memory.	[47]
*S. lavandulaefolia* (25 and 50 μL)	Healthy individuals, PC, DB, balanced, crossover trail (n = 24)	CDR & Bond-Lader mood scale	Improvement on the speed of memory and secondary memory.	[48]
Tofu	Honolulu-Asia Aging Study (n = 3734)	All task included in Cognitive abilities screening instrument(CASI)	Higher midlife tofu consumption was independently associated with indicators of cognitive impairment and brain atrophy in late life.	[49]
Dietary phytoestrogens	Cross-sectional study (n = 301)	Functions like, memory, processing capacity, speed and executive function	High lignan intake improves capacity, speed and executive function.	[50]

DB = Double blind; SB = Single blind; PC = Placebo controlled; R = Randomized; M = Multicentre; ADAS-Cog 11 = 11-item Alzheimer’s disease Assessment Scale-Cognitive subscale; CASI = Cognitive abilities screening instrument; MMSE = Mini-mental state examination; CDR =Clinical dementia rating; ADL = Activities of daily living; CERAD = Consortium to Establish a Registry for AD Assessment Packet; K-CVLT = Korean version of the California Verbal Learning Test; SOPT = Self-Ordered Pointing Test; Auditory Verbal Learning Test (AVLT); DAT = Divided Attention Task; WAIS = Wechsler Adult Intelligence Scale; AVLT = Auditory Verbal Learning Test; Nurnberger NAB = Alters-Beobachtungsskala; SKT = Syndrome Kurz Test; CGI = Clinical Global Impression; NAI-NAA = Nuremberg Gerontopsychological Rating Scale for Activities of Daily Living; WJCAT III = Woodcock-Johnson Cognitive Abilities Test III.

Similar trends were apparent in study 2 (GAL-USA-5; n = 118); in patients with mild to moderate AD who had exhibited cognitive benefits from up to 5 months galantamine treatment, continuing therapy reinforced previously achieved benefit, whereas patients in whom galantamine was discontinued, the natural progression of AD was apparent [51]. It is important to consider long-term effects in patients with AD, not only in patients receiving treatment, but also in subjects in whom therapy has been discontinued. One study evaluated MMSE scores for up to 7 years to identify the long-term effects of galantamine on cognitive function in patients with AD, using both clinical data and epidemiological modeling. Patients continuing galantamine treatment and those who stopped were also considered in this study. The results showed that patients with mild-to-moderate AD who received long-term galantamine treatment exhibited an attenuated decline in cognitive function, as assessed by the MMSE, compared with decline predicted in the absence of treatment. Furthermore, patients who stopped treatment experienced subsequent cognitive decline at a rate similar to that predicted for untreated patients [52]. In an intervention review, ten clinical trials consisting primarily of mildly to moderately impaired outpatients (n = 6,805) were included in the analysis. Data from earlier studies were analyzed, and the authors concluded that galantamine showed positive effects for trials of 3–6 month duration. Moreover, the safety of the galantamine profile in patients with AD was similar to that of other cholinesterase inhibitors with respect to cholinergically mediated gastrointestinal symptoms. Galantamine use is not recommended for patients with mild cognitive impairment (MCI) due to its association with an excess death rate [53]. Cholinesterase inhibitors have potentially troubling side effects such as hepatotoxicity, gastrointestinal symptoms, insomnia, and muscle weakness [54,55,56]. Galantamine seems to overcome the troublesome side effects of nausea, vomiting, dizziness, and anorexia [13,14,15,17,18,19].

### 2.2. Huperzine A from Huperzia serrata

Huperzine A (HupA, Figure 1B) is a natural cholinesterase inhibitor obtained from the Chinese herb *Huperzia serrata* (Thunb. Ex Murray) Trev. a member of the Huperziaceae family. Huperzine is known worldwide as a medicinal plant since Chinese scientists discovered huperzine A [57]. It has been widely shown to reverse or attenuate loss of cognition in several behavioral models and different animal species including non-human primates [58]. HupA is the most-promising drug candidate with potent anticholinesterase effects and is a licensed anti-AD drug in China [59]. HupA crosses the blood-brain barrier, has oral bioavailability, long duration of AChE inhibitory action [60], and it is estimated that 100,000 people have been treated with HupA in China [61]. 

In one of the earlier randomized, double-blind clinical trials conducted on 28 individuals with AD, patients either received a total daily dose of 0.4 mg of HupA or a placebo. The outcomes were assessed with the MMSE, HDS, WMS, ED and ADL tests. HupA treatment improved memory test scores compared to that of placebo [20]. HupA at 0.4 mg/day was subsequently tried in patients diagnosed with AD or VaD. Using the MQ test, a popular memory test in China, the treatment group improved by 9.37 points, as compared to 1.9 points in the control group [21]. In another clinical trial, 60 patients were treated for 60 days with 0.4 mg/d HupA. The main aim of that study was to examine the effect of the drug formulation on treatment. One treatment group was administered tablets and the other treatment group received capsules. Although mild adverse events (insomnia, nausea, and vomiting) were reported in both groups, as measured by the MMSE and MQ tests, the treatments were revealed to be effective. In addition, the capsules were more effective than the tablets [62]. After initial studies, the number of participants increased in following studies. A total of 202 patients from 15 centers nationwide with a diagnosis of possible or probable AD were randomly divided into two groups: HupA alpha group (n = 100, given HupA alpha 400 µg/day for 12 weeks) and a placebo group (n = 102). Different scales were utilized to evaluate the cognitive function. As a result, HupA alpha remarkably improved cognition, behavior, ADLs, and mood of patients with AD as assessed by the ADAS-Cog 11 (4.6 points), MMSE (2.7 points), behavior and mood by the ADAS-non-Cog (1.5 point), ADL (2.4 points) compared with those of the baseline data [22]. In another multicenter, randomized trial on patients with mild to moderate AD, in which 210 individuals were randomized to receive placebo (n = 70) or HupA [200 μg bid (n = 70) or 400 μg bid (n = 70)] for at least 16 weeks, 177 subjects completed the treatment phase. No difference was observed on the ADAS-Cog 11 following administration of 200 μg bid HupA at week 16 than that of 200 μg bid placebo in primary analyses. The increased dose of 400 μg HupA bid showed 2.27 and 1.92 point improvements in the ADAS-Cog 11 at 11 and 16 weeks, respectively. It was concluded that 200 μg bid HupA has no demonstrable cognitive effects in patients with mild to moderate AD [23]. A recent randomized double-blind, placebo-controlled study of 78 patients with mild to moderate VaD were randomized to receive vitamin C (100 mg bid) as a placebo (n = 39) or HupA (0.1 mg bid) (n = 39) for 12 consecutive weeks. Cognitive enhancing effects were assessed by the MMSE, Clinical Dementia Rating (CDR), and ADL scores. HupA showed significant improvement compared to that of the placebo-controlled group without any serious adverse effects [24].

### 2.3. Bacosides from Bacopa monnieri

*Bacopa monnieri* (L.), a plant belonging to the Scrophulariaceae family, has been used in the traditional system of Ayurvedic medicine, either alone or in combination with other herbs to improve intelligence and memory [63]. It also has other medicinal properties such as, anti-inflammatory, analgesic, antipyretic, sedative, and antiepileptic effects [64]. This plant is called Brahmi in Hindi and water hyssop in English. The major active constituents are the steroidal saponins, bacosides A and B [65]. Besides D-mannitol, betulic acid, sitosteron, and stigmasterols [65], bacoside A (Figure 1C) is considered responsible for the memory enhancing effect of this plant [66]. Extract of *B. monnieri* improves motor learning [67], acquisition, consolidation, and retention of memory in rats [63]; alleviates both anterograde and retrograde amnesia induced by scopolamine injection [68]; attenuates neurotoxin and immobilization stress [69]; provide protection from phenytoin-induced cognition deficit [70]; and shows potential neurotrophic and neuroprotectant activities against ethylcholine mustard aziridinium ion-induced behavioral deficits [71]. The probable mechanism responsible for the cognitive enhancing activities include modulation of the cholinergic system and/or antioxidant effects [69], choline acetylase activity [72], acetylcholine release and muscarinic cholinergic receptor binding [73], potent antioxidant effects [74,75], and free-radical scavenging activity [76]. Bacosides (450 mg) were safe in phase I clinical studies involving male healthy human volunteers following both single and chronic treatment [77]. Initial uncontrolled trials reported learning and memory enhancing effects of bacopa with long-term dosing in children [78] and in patients with anxiety neurosis [79]. Double-blind placebo-controlled investigations were performed to further evaluate the true effects of *B. monnieri*. Neuropsychological tests conducted before and 2 h after administration of 300 mg *B. monnieri* did not show any effect on cognitive functioning in healthy human subjects; thus, there was no effect of an acute dose of *B. monnieri* [80]. To identify chronic effects, healthy individuals 18–60 years participated to investigate the memory enhancing effect of *B. monniera* (KeenMind). Forty-six participants were randomly allocated to either placebo or *B. monniera* treatment groups. Batteries of well-validated neuropsychological tests were utilized for checking efficacy. After 90 days of *B. monniera* (KeenMind) administration, a significant improvement was reported for information processing, memory consolidation, as well as a significant reduction in anxiety [26]. In anotherdouble-blind randomized, placebo control clinical study, 76 adults with an age range of 40–65 years were dosed with *B. monniera*, and memory functions were tested along with levels of anxiety. Three testing sessions, one prior to the trial, one after 3 months of the trial, and one 6 weeks after completion of the trial were conducted to determine the effect of *B. monniera* on human memory. *B. monniera* showed significant effects on a test for retaining new information and decreased the rate of forgetting newly acquired information. Other parameters such as tasks assessing attention, verbal, and visual short-term memory, a questionnaire that measures everyday memory function, and anxiety levels were unaffected [81]. In a similar manner, another double-blind placebo-controlled independent group design was carried out to assess spatial working memory. In total, 107 healthy participants were recruited, and neuropsychological testing using the Cognitive Drug Research (CDR) cognitive assessment system was conducted at baseline and after 90 days of treatment with a *B. monniera* extract (2 × 150 mg KeenMind) or placebo. The results showed that the *B. monniera* product significantly improved spatial working memory and reduced the number of false positives during a rapid visual information processing task [27]. 

A randomized, double-blind, placebo-controlled clinical trial with a placebo run-in for 6 weeks and a treatment period of 12 weeks was carried out to observe the potential of *B. monnieri* for enhancing cognitive deterioration observed during aging. Fifty-four participants, 65 years or older without clinical signs of dementia, were recruited and randomized to receive Bacopa or placebo. A standardized *B. monnieri* extract of 300 mg/day or a similar placebo tablet was administered orally for 12 weeks. Bacopa participants had enhanced auditory verbal learning test scores and delayed word recall memory scores compared to those administered the placebo. Ability to ignore irrelevant information as assessed by the Stroop test improved significantly with bacopa treatment, but it was unchanged with placebo. No effect on the Divided Attention Task, or the Wechsler Adult Intelligence Scale letter-digit test of immediate working memory was observed. This study provided further evidence that *B. monnieri* has potential for safely enhancing cognitive performance during aging [28]. BacoMind^®^ (extract of *B. monnieri*) at a dose of 300 mg/day or an identical placebo was evaluated in 98 healthy participants >55 years of age from the general population. BacoMind significantly improved verbal learning, memory acquisition, and retention in a healthy elderly population with some gastrointestinal tract side effects [82]. Moreover, apart from the cognitive enhancing effect of *B. monnieri* in elderly patients, a recent study of this plant showed that it is effective in patients with AD. This study was an open label, prospective, uncontrolled, non-randomized trial performed in newly diagnosed patients with AD between 60 and 65 years of age. MMSE scores were recorded for all patients before and after administration of 300 mg Bacognize^®^ (standardized extract of *B. monnieri*),orally twice per day for 6 months. *B. monnieri* produced statistically significant improvements in various components on the MMSE, including orientation of time, place and person, attention, and in the language component in terms of reading, writing, and comprehension at the end of the trial [25]. *B. monnieri* (CDRI08 300 mg/day) was selected as a candidate to reduce cognitive aging in the Australian Research Council longevity intervention (ARCLI). ARCLI is a randomized, placebo-controlled, double-blind, three-arm clinical trial in which 465 healthy elderly participants will be randomized to receive an extract of *B. monnieri* (CDRI08 300 mg/day), pycnogenol (150 mg/day), or placebo daily for 12 months. Participants will be tested at baseline and then at 3, 6, and 12 months post-randomization on a wide battery of cognitive, neuropsychological, and mood measures, as well as cardiovascular, biochemical, and genetic assessments. The primary aim is to investigate the effects of these supplements on cognitive performance. The secondary aim is to explore the time-course of cognitive enhancement as well as potential cardiovascular and biochemical mechanisms underpinning cognitive enhancement over the 12 months of administration. Results from ARCLI may help develop novel preventive health practices and nutritional/pharmacological targets in the elderly for cognitive and brain health [83].

### 2.4. Ginkgolides from Ginkgo Biloba

*Ginkgo biloba*, family Ginkgoaceae, is a living fossil tree having undergone little evolutionary change over almost 200 million years. It is mainly cultivated for its nuts and leaves. The most important constituents of standardized extracts of dried *G. biloba* leaves are flavone glycosides (quercetin, kaempferol, and isorhamnetin) and terpene lactones (ginkgolides and bilobalide) (Figure 1D,E) [84,85]. *G. biloba* extract is among the most commonly prescribed medications in Germany and France [86] and is one of the most widely used herbal dietary supplements in the United States [87]. A standardized commercial *G. biloba* extract called EGb 761 is popular and contains various biologically active constituents. This standardized extract is available in several commercial forms for treating or preventing claudication, cognitive decline, dementia, and cerebral insufficiency. This extract increases the rate of acetylcholine turnover and stimulates binding activity of ligands to muscarinic receptors in the hippocampus [88]. *G. biloba* cures cognitive deficits and dementia [31,89] and also has antioxidant and free radical scavenging activity in animals [90,91,92]. The multiple effects *G. biloba* can be attributed to the different components of the extract, which may act independently or synergistically.

In an initial randomized, double-blind, placebo controlled, multi-center study conducted in patients with dementia of the Alzheimer type and multi-infarct dementia, 216 patients were randomized and received EGb 761 at a dose of 240 mg or placebo once daily for 24 weeks. The Syndrom–Kurz test (SKT) for attention and memory, the Clinical Global Impressions (CGI) scale for psychopathological assessment, and the Nurnberger Alters-Beobachtungsskala (NAB) test were used for behavioral assessment of ADLS. The results showed that EGb 761 was effective in patients with dementia of the Alzheimer type and multi-infarct dementia [31]. In individuals between 55 and 86 years of age with no history of significant neurocognitive dysfunction, EGb 761 (180 mg/day) daily for 6 weeks significantly improved task assessing speed for processing abilities (*i.e.*, Stroop color and word test color-naming task) by the end of treatment compared to those who received placebo. These findings suggest that short-term use of EGb 761 might improve cognitive processes in older adults [33]. In a 24 week randomized, double-blind, placebo-controlled, parallel-group, multicenter study conducted in elderly patients with dementia (AD or VaD) or age-associated memory impairment (AAMI), 214 participants were randomly allocated to receive either EGb 761 (240 mg/day or 160 mg/day) or placebo. Primary outcome measures in this study were the SKT, CGI, and the Nuremberg Gerontopsychological Rating Scale for ADLs (NAI-NAA; behavioral functioning). As a result, no significant differences were observed between the ginkgo and placebo group; thus, the authors concluded that EGb 761 is not beneficial for patients with dementia or AAMI [93]. In a randomized, double-blind, placebo-controlled clinical trial conducted between 2000 and 2008 in the US, 3,069 community volunteers aged 75 years or older with normal cognition (n = 2587) or MCI (n = 482) were assessed every 6 months for the incidence of dementia. *G. biloba* at a dose of 120 mg (n = 1,545) or placebo (n = 1,524) were given twice daily. The overall dementia rate was 3.3 per 100 person-years in participants assigned to the *G. biloba* group and 2.9 per 100 person-years in the placebo group. *G. biloba* at 120 mg twice per day was not effective for reducing either the overall incidence rate of dementia or AD incidence in elderly individuals with normal cognition or those with MCI [94]. *G. biloba* has also been evaluated in patients with AD and VaD. A total of 395 patients aged 50 years or above with dementia with neuropsychiatric features were treated with EGb 761 (240 mg/day) or placebo for 22 weeks. Efficacy was assessed by the SKT test battery (primary outcome measure), Verbal Fluency Test, Clock-Drawing Test, NPI, Hamilton Rating Scale for Depression, and the Gottfries-Brane-Steen Scale. Results from the various batteries of test inferred that EGb 761 treatment benefited patients with AD and VaD [95]. In a recent study, middle-aged healthy volunteers (n = 188), age 45–56 years were randomized to receive EGb 761 (240 mg once daily) or placebo for 6 weeks. Memory performance was evaluated using the demanding standardized free recall paradigm (list of appointments) and a less demanding standardized recognition test (driving-route). After 6 weeks, EGb 761-treated subjects improved significantly in recall, *i.e.* the number of correctly recalled appointments, with no benefit on the recognition test. Free recall of appointment requires high demands on self-initiated retrieval of learned material and is sensitive to normal aging. In this same regard, EGb 761 also shows benefits on demanding cognitive tasks [96]. A recent multi-center, double-blind, randomized, placebo-controlled, 24-week trial with 410 outpatients was conducted to demonstrate the efficacy and safety of 240 mg EGb 761 in patients with mild to moderate dementia (AD or VaD) associated with neuropsychiatric symptoms. Primary outcomes were the changes from baseline to week 24 on the SKT and the NPI total score. The ADCS Clinical Global Impression of Change, Verbal Fluency Test, and Activities of Daily Living International Scale were secondary outcome measures. Patients treated with EGb 761 showed improvement in both SKT and NPI scores compared to those administered the placebo and also improved on the secondary outcome measures. Thus, EGb 761 at 240 mg was found to be safe and effective [97].

### 2.5. Asiatic Acid from Centella asiatica

**Figure 2:**
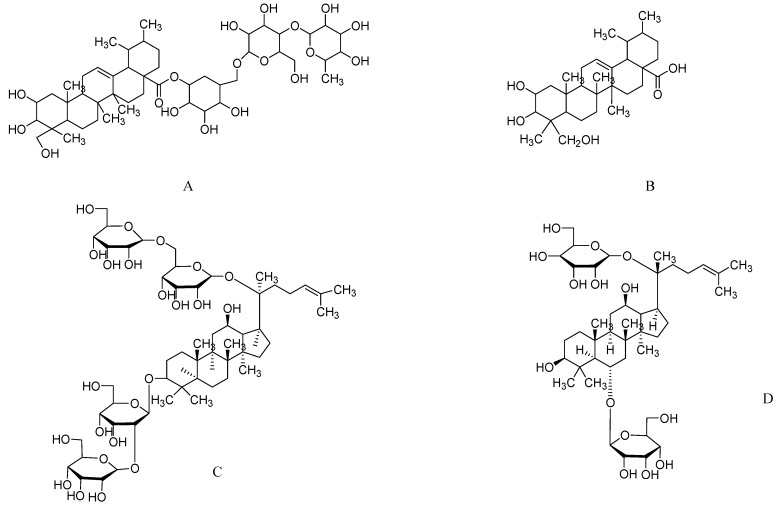
The molecular structures of asiaticoside (**A**), asiatic acid (**B**), ginsenoside Rb1(**C**), and ginsenoside Rg1 (**D**).

*Centella asiatica (C. asiatica)* (L.), belonging to the family Apiaceae, is also known as *Mandookaparni* in the Indian system of medicine. In the Indian literature, this herb is reported to have central nervous system (CNS) effects such as nerve tonic, rejuvenant, sedative, and tranquilizer effects and is reputed to increase intelligence and memory [98]. In the USA and other Western countries it is sold as the dietary supplement as “gotu kola” [99]. *C. asiatica* has a reputation to restore declined cognitive functions in patients with TCM and in animal models of learning and memory. Triterpene saponosides, including asiatic acid (Figure 2B), madecassic acid (6-hydroxy-asiatic acid), asiaticoside (Figure 2B), madecassoside, madasiatic acid, betulinic acid, thankunic acid, and isothankunic acid are major chemical compounds found in this plant [100,101]. Moreover, some other triterpenes such as brahmic acid, centellin, centellicin, asiaticin, bayogenin, and terminolic acid are also found. It has been used in ayurvedic preparations either in a fresh or extract form [102]. Of the aqueous, methanolic, and chloroform extracts of *C. asiatica*, only the aqueous extract of the entire plant shows improvement in learning and memory [103,104,105], inhibition of AChE activity [103,106], improved dendritic arborization of the amygdala and hippocampus [103,107,108], reduced levels of β-amyloid plaques in the hippocampus [109], decreased oxidative stress [104,105], prevention of radiation-induced behavioral changes during clinical radiotherapy [110], and improved D-galactose induced behavioral, biochemical, and mitochondrial dysfunctions in animals [111]. 

Asiaticoside, the active constituent of *C. asiatica* has been reported as a dementia-treatmenting agent and cognitive enhancer [112]. Asiaticoside has therapeutic value against β-amyloid neurotoxicity [113]. Although many *in vivo* studies have been carried out on CNS-related effects of *C. asiatica*, a literature survey revealed the presence of only a limited number of clinical studies on this species. A double blind study in children with mental disabilities showed that *C. asiatica* administration for 3 and 6months resulted in significant improvements. In a randomized, double-blind, and placebo-controlled, clinical study, 28 healthy and elderly volunteers, consisting of four men and 24 women with an average age of 65.05 ± 3.56 years, were administered a capsulated aqueous extract of *C. asiatica* standardized to contain 29.9 mg/g tannic acid, 1.09 mg/g asiaticoside, and 48.89 mg/g asiatic acid at doses of 250, 500, and 750 mg over 2 months. Cognitive performance was evaluated by a variety of parameters using a computer-assisted technique Among the dose ranges, only the highest dose of the *C. asiatica* extract showed a cognitive enhancing effect [35]. In a similar study, cognitive performance following administration of capsulated *C. asiatica* extract was evaluated in 41 (22 women and 19 men) middle-aged healthy subjects using the Woodcock-Johnson Cognitive Abilities Test III, prior to the trial (baseline), and 40, 60, and 90 days after treatment. The capsule was given at various doses, ranging from 3 to 4 g once per day, for 2 months. The cognitive test results indicated that the extract of *C. asiatica* resulted in a remarkably positive influence in all subjects [114]. A study aimed to manage cognitive functioning in an elderly population (≥65 years) with MCI and other age-related problems was conducted with 60 patients. Extract of *C. asiatica* was prescribed at a dosage of 500 mg twice per day (1,000 mg daily) for 6 months. The diagnostic tools used in this study were the MMSE, ADL, ADL-IL, and Yesavage Geriatric Depression Scale. The outcomes showed improvement in depression and other age-related conditions such as hypertension, insomnia, loss of appetite, and constipation. The mean MMSE scores improved significantly after 6 months of *C. asiatica* administration in elderly subjects with MCI. This finding indicates that *C. asiatica* is clinically useful in patients suffering from MCI [115].

### 2.6. Ginsenosides from Ginseng

In recent decades, scientists in Asian and Western countries have been paying a great deal of attention to research on ginseng commonly known as *Panax ginseng* C.A Meyer. More than 200 ginsenosides and non-saponin constituents have been isolated and identified from ginseng. Among the multitude of activities shown by ginseng, anti-aging and particularly the nootropic effect are noticeable [116]. Among the 30 major ginsenosides, Rb1 and Rg1 (Figure 2C,D) are the main active ingredients of *Panax* species [117]. Rg1 improves learning and memory in normal rats and mice. The nootropic signaling pathway has also been assessed in normal rats, and the Rg1-induced signaling pathway is similar to memory formation that occurs in mammals, suggesting that Rg1 may have a potential effect on increasing intellectual capacity in normal people [118]. Ginsenoside Rh2 recovers the scopolamine-induced reduction in long-term potentiation in the hippocampal CA1 area [119]. Rb1 enhances the stimulatory effect of neurite outgrowth [120], promotes neurotransmitter release by modulating phosphorylation of synapsins through a cAMP-dependent protein kinase pathway [121], and upregulates cell genesis in the dentate gyrus (DG) and CA3 hippocampal subregions [122]. The use of herbal remedies, particularly ginseng, has become increasingly popular for improving cognitive performance in recent years. Several studies have indicated that the administration of ginseng may improve learning and memory in healthy volunteers [36,123]. 

In a related study, the single dose effects of ginseng on aspects of mood and cognitive performance in healthy and young adult volunteers were assessed via a randomized placebo-controlled, double-blind, balanced, cross-over design. Twenty participants received a random treatment of 400 mg ginseng and a placebo with a 7-day wash-out period between treatments. Cognitive testing comprised completion of the CDR computerized assessment battery and two serial subtraction mental arithmetic tasks. Furthermore, Bond-Lader visual analogue scales were used to assess mood. The results clearly showed that ginseng improves the speed of performing memory tasks and the accuracy of attentional tasks [36]. In a study combining *G. biloba* and *P. ginseng*, which evaluated memory enhancing activity, the cognitive functioning effects of capsules containing 60 mg of a standardized extract of *G. biloba* (GK501) and 100 mg of a standardized extract of *P. ginseng* (G115) were assessed in healthy middle-aged volunteers. Additionally, a double blind, placebo controlled trial, involving 256 middle-aged volunteers for 14 weeks, with a parallel group and repeated assessment in multiple centers was conducted with two dosing regimens of 160 mg bid and 320 mg od. On various study days (weeks 0, 4, 8, 12, and 14) the volunteers performed a selection of attention and memory tests from the CDR computerized cognitive assessment system prior to morning dosing and, again, 1, 3, and 6 h later. They also completed questionnaires about mood states, quality of life, and sleep quality. The ginkgo/ginseng combination significantly improved the Index of Memory Quality. An improvement of 7.5% and reflected improvements in a number of different memory aspects, including long-term memory and working were observed. This memory enhancement was seen throughout the 12-week dosing period and even after a 2-week washout period. These results are the first substantial demonstration of memory improvements in healthy middle-aged volunteers produced by a combination of *P. ginseng* and *G. biloba* [34]. Based on these studies, it is evident that single dose administration of ginseng can improve certain aspects of cognitive performance and mood in healthy young volunteers. Another double-blind, placebo controlled, balanced, cross-over design study, aimed to investigate the effect of acute administration of 400 mg of a standardized *P. ginseng* extract (G115, Pharmaton SA) on mood and cognitive performance, was carried out. This study involved 30 healthy young volunteers who received 400 mg ginseng and a matching inert placebo in a counter balanced order, with a 7-day washout period between treatments. Cognitive performance and mood were assessed at 90 minutes after drug ingestion. Ginseng improved attention speed, indicating a beneficial effect on a participant’s ability to allocate attentional processes to a particular task. Ginseng also improved tasks associated with episodic memory performance at 1, 2.5, 4, and 6 h post-ingestion [37]. Over the last decade, Asian ginseng (*P. ginseng*) has been shown to improve aspects of human cognitive function. The ginsenoside profile of *P. ginseng* is different from that of American ginseng (*P. quinquefolius*). In this study, neurocognitive properties of standardized extract of *P. quinquefolius* (Cereboost™) were tested in humans. This randomized, double-blind, placebo-controlled, crossover trial involved 32 healthy young adults. Following administration, parameters such as mood and neurocognitive effects at 1, 3, and 6 h after dosing (100, 200, and 400 mg) with Cereboost™ (*P. quinquefolius* standardized to 10.65% ginsenosides) were assessed. The results indicated that *P. quinquefolius* significantly improved working memory performance at all testing times. The 100 mg dose significantly improved choice reaction time accuracy and calmness. These effects were distinct from those shown by Asian ginseng and indicate that psychopharmacological properties depend critically on the ginsenoside profile [38]. However, there is a lack of research on the cognitive and mood effects of repeated ginseng ingestion. Therefore, the next placebo-controlled, double-blind, randomized, crossover was utilized to assess the effects of *P. ginseng* (G115) on subjective mood and aspects of working memory processes, following single dose and subchronic (7 day) ingestion in healthy volunteers. This study involved 30 volunteers (mean age, 22.87 ± 4.01 years) who received treatment (200, 400 mg, or placebo) for 8 days in a counter-balanced order, with a wash out period of 6 days. Evaluations were performed on days 1 and 8 of each treatment period, at pre-dose and at 1, 2.5, and 4 h post-dose. The results confirmed that an acute dose of *P. ginseng* (G115) modulates cognitive function and mood in young healthy volunteers; however, the study revealed no effects following a 7-day dosing regimen [39]. HT1001™, a proprietary North American ginseng extract containing known levels of active ginsenosides, may improve cognitive function. In that study, HT1001 was characterized for its effect on working memory in a group of stable individuals suffering from schizophrenia. In a double-blind, placebo-controlled study design, 64 patients satisfying the Diagnostic and Statistical Manual-IV criteria for schizophrenia randomly received either HT100 or placebo for 4 weeks. Verbal working memory and visual working memory were assessed at baseline and again at the end of the treatment phase using the Letter-Number Span Test and Visual Pattern Test, respectively. HT1001 significantly improved visual working memory as compared to that in the placebo group. Furthermore, extrapyramidal symptoms were decreased significantly 4 weeks post-treatment in the HT1001 group compared to that in the placebo group. These results provide a solid base for further investigation of HT1001 as an adjunct therapy in patients with schizophrenia for improving working memory and reducing medication-related side effects [40].

### 2.7. Epigallocatechin-3-gallate from Green Tea

**Figure 3:**
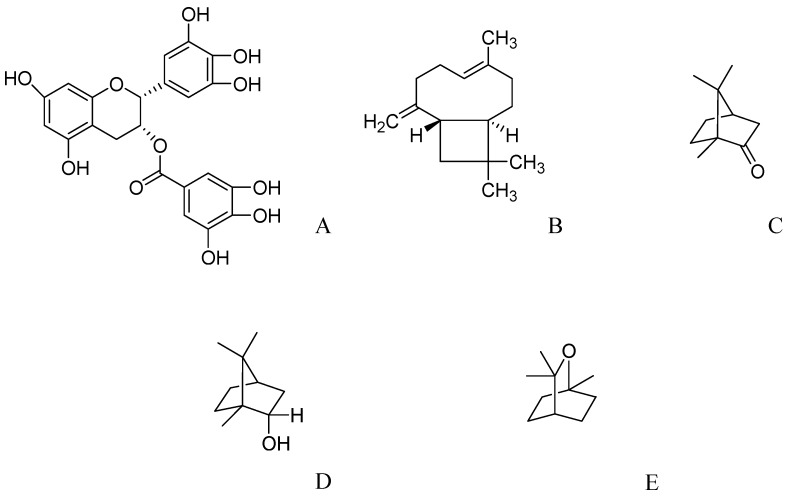
The molecular structures of epigallocatechin-3-gallate (**A**), caryophyllen (**B**), camphor (**C**), borneol (**D**), and 1,8-cineol (**E**).

Green tea, obtained from *Camellia sinensis*, is rich in polyphenolic compounds as active constituents which are reported to have diverse biological effects, including anti-inflammatory, anticancer, antidiabetic, antihypertensive [124], and neuroprotective effects [125,126,127]. The major polyphenolic constituents in green tea are epigallocatechin-3-gallate (EGCG), epicatechin (−) epigallocatechin and (−) epicatechin-gallate. EGCG (Figure 3A) is the prominent and most active component of green tea catechins [128,129]. It acts as an antioxidant [130] and attenuates lipid peroxidation caused by various forms of free radicals in biological systems [131]. GTPP has also been reported for their cognition enhancing ability [132] and for abating oxidative changes associated with aging [133]. Green tea may possess potent neuroprotection and amyloid precursor protein processing activities that may lead to cognitive enhancement. Green tea catechins potential in aging-impaired cognition and neurodegenerative diseases have been reviewed [134], and it might be used for disease modifying therapy in combination with other compounds. In recent years, green tea has been explored in clinical trials for learning and memory-related disorders, indicating its potential for improving learning and memory [135]. In a randomized, double-blind, placebo-controlled study, a combination of green tea extract and L-theanine (LGNC-07) on memory and attention in subjects with MCI was investigated. Ninety-one patients with MCI whose MMSE-K scores were 21–26 and who were in either stage 2 or 3 on the Global Deterioration Scale were selected for this study. The treatment schedule consisted of 16 weeks LGNC-07 (1,680 mg) administration for the treatment group (13 men, 32 women; 57.58 ± 9.45 years) and an equivalent amount of maltodextrin and lactose for the placebo group (12 men, 34 women; 56.28 ± 9.92 years). The effect of LGNC-07 on memory and attention was evaluated by electroencephalography and neuropsychological tests (Rey-Kim memory test and Stroop color-word test). Baseline severity was used to evaluate treatment response based on the degree of impairment. LGNC-07 marginally improved delayed recognition on the Rey-Kim memory test. Further analyses showed that LGNC-07 improved selective attention and memory by significantly increasing the Rey-Kim memory quotient and word reading in subjects with MMSE-K scores of 21–23. After a single LGNC-07 dose, electroencephalograms were recorded from 24 randomly selected subjects hourly for 3 h in the eye-open, eye-closed, and reading states. Data revealed that brain theta waves (indicator of cognitive alertness) increased significantly in the temporal, frontal, parietal, and occipital areas after 3 h in the eye-open and reading states. Therefore, these results indicate that LGNC-07 has potential therapeutic application for cognitive improvement [41]. 

In some clinical studies, consumption of green tea was assessed in older aged subjects, and their cognitive functions were explored. These trials were conducted based on a cross-sectional design and involved volunteers of 55–80 years. Data from these trials disclosed that tea consumers had better performance on the modified-MMSE [42,43], Trail Making Test-A, modified- Digit Symbol Test, and modified Block Design test compared to those of non-consumers [136]. Cognitive impairment was associated with a lower frequency of tea consumption in men but not women [137]. Total tea (green along with black/oolong tea) consumption improves performances on global cognition memory, executive function, and information processing speed [44].

### 2.8. Essential Oils from Salvia Species

**Figure 4:**
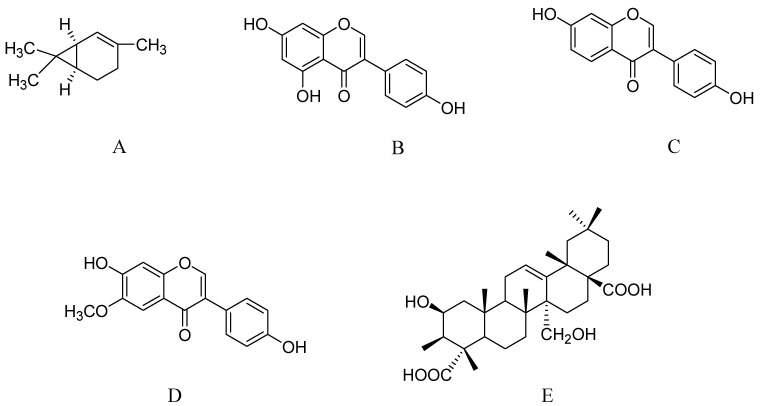
Molecular structures of carene (**A**), genistein (**B**), daidzein (**C**), glycitein (**D**), and presenegenin (**E**).

Most pharmacological research on *Salvia* has been conducted with the essential oils of *Salvia officinalis* and *Salvia lavendulifolia*, and a number of promising pharmacological results have been published. Long ago these plant species were used to treat “weak brain” [138]. The components of the essential oil vary in different *Salvia* species [139]. The oil of *S. officinalis* is rich in α-caryophyllene (23.2%), camphor (11.0%), and borneol (8.7%), (Figure 3B–D), whereas camphor,1,8-cineole, and 2-carene (Figure 3E and Figure 4A) are abundant in the oil of *S. lavendulaefolia* [140]. *S. lavandulaefolia* and *S. officinalis* purpurea oils have apparent dual cholinergic activity, as they are active in both AChE and pseudocholinesterase [139]. *Salvia* (commonly called sage) is an important genus in the family Lamiaceae, consisting of around 900 species. Species of *Salvia* are cultivated worldwide for use in folk medicine and for culinary purposes. *Salvia* species have various biological activities in the CNS such as analgesic, memory enhancing [139], antiparkinsonian, sedative and hypnotic, hallucinogenic, skeletal muscle relaxant, and anticonvulsant activities, as well as the inhibition of ethanol and morphine withdrawal syndrome [141]. 

Based on TCM, *S. officinalis* has *in vitro* cholinergic binding properties and modulates mood and cognitive performance in humans. Therefore, *S. officinalis* might potentially provide a novel natural treatment for AD. A double blind, randomized and placebo-controlled trial was conducted to determine the efficacy and safety of an *S. officinalis* extract using a fixed dose of 60 drops/day for 4 months in patients with mild to moderate AD. Subjects for the trial were 65–80 years (n = 42, 18 women) and were selected based on the cognitive subscale of ADAS-Cog 11 and the CDR. A fixed dose of *S. officinalis* extract was administered in a random manner following the same schedule as that of the placebo. At 4 months, the *S. officinalis* extract produced significantly better outcomes for cognitive functions than those of the placebo. No significant differences were observed between the two groups in terms of observed side effects except agitation, which appeared to be more frequent in the placebo group. Hence, these results clearly showed the efficacy of *S. officinalis* extract for managing mild to moderate AD [45]. Another recent randomized, placebo-controlled, double-blind, balanced, five-period crossover controlled trial investigated the acute effects on cognitive performance in 20 older adults (>65 years of age, mean, 72.95 years) who were administered a standardized extract of *S. officinalis*. The *S. officinalis* extract was dosed in four active parts (167, 333, 666, and 1,332 mg) and a placebo with a 7-day wash-out period between visits. Assessment involved completion of the CDR computerized assessment battery at 1, 2.5, 4, and 6 h post-treatment. The results showed that a 333 mg dose of *Salvia* extract significantly enhanced secondary memory performance at all testing times compared with that in the placebo group. The same effect was seen to a lesser extent in the other doses. There was also a significant improvement on accuracy of attention following the 333 mg dose. The overall results indicate a dose-related effect on memory consolidation [46]. It has been speculated that the active compounds in *S. officinalis* might also be present in the aromas; thus, even the aroma might produce a similar cognitive enhancing effect. Therefore, in an independent experimental design, the *S. officinalis* aroma, the *S. lavandulaefolia* aroma, and no aroma were tested in 135 healthy volunteers for their effects on cognition and mood. Parameters such as cognitive performance were assessed via the CDR system, and mood was assessed by Bond-Lader Mood scales on three dimensions before and after the cognitive tasks. Data obtained revealed that the *S. officinalis* aroma group performed significantly better than that in the control group on the quality of memory and secondary memory primary outcome factors. The aroma treatment significantly increased mood alertness as compared to that in the control group. These findings suggest that the aromas of *Salvia* species essential oils have a positive effect on cognition [47].

Another important member of the sage family is *S. lavandulaefolia*, which also has a long history of being used as a memory-enhancing agent due to its cholinergic properties that may potentially be relevant to amelioration of cognitive deficits associated with AD. A placebo-controlled, double-blind, balanced, crossover design was used to test the effect of *S. lavandulaefolia* on cognition and mood. In that study, 24 volunteers received a single dose of 25 or 50 μL of a standardized essential oil of *S. lavandulaefolia* separated by a 7 day washout period. Cognitive performance was assessed pre and post-dosing at 1, 2.5, *4*, and 6 h using the CDR computerized test, and mood was assessed by Bond-Lader visual analogue scales. The results showed that administration of 25 and 50 μL *S. lavandulaefolia*resulted in a consistent improvement on memory speed. Improvement was also seen on the secondary memory factor at a dose of 25 μL. Mood was consistently enhanced following the 50 μL dose, and elevated calmness was observed following the 25 μL dose. These results further demonstrate that *Salvia* can acutely modulate mood and cognition in healthy young adults [48].

### 2.9. Phytoestrogens from Tofu

Phytoestrogens are chemicals found naturally in certain plants that mimic the action of estrogen. They can be found in a wide variety of foods, most notably soya products such as tofu and tempe (a fermented whole soybean product). Isoflavones are the most widely know group of phytoestrogens and are present in berries, wine, grains, and nuts but are most abundantly present in soybeans and other legumes [142]. Soy products such as soy milk, tofu, and tempe, constitute 60% of processed foods [143], and a report published in 2009 by the United Soybean Board stated that 84% of US consumers perceive soy as healthy and 32% purposefully consume soy products at least once per month [144]. Dietary soy phytoestrogens enhance visual spatial memory in female rats but inhibit it in males [145]. Recently published data also indicate the AChE activity of Chinese sufu (fermented tofu) [146]. 

An initial clinical study from the Honolulu-Asia Aging Study (HAAS) was conducted with participants (n = 3,734) from a longitudinal study established in 1965 for research on heart disease, stroke, and cancer. Participants were interviewed from the years 1965 to 1967 and 1971 to 1974 to obtain a detailed record of their consumption of food. Based on this record, a four-level composite intake index was prepared as high-high” consumers, “low-low” consumers, “low”, and “high” consumers. Cognitive functioning was tested in participants aged 71–93 years. It was evident that higher midlife tofu consumption resulted in poor cognitive test performance, enlargement of ventricles, and lower brain weight. A similar association between midlife tofu intake and poor late-life cognitive test scores was also observed among wives of cohort members, using the husband’s answers to food frequency questions as a proxy for the wife’s consumption. Conclusively higher midlife tofu consumption was independently associated with indicators of cognitive impairment and brain atrophy in late life [49]. Another cross-sectional study reported that the effect of dietary phytoestrogens on aging is associated with a decline in cognitive function. That study involved Dutch women volunteers (n = 301/60–75 years). The Food-Frequency Questionnaire was conducted to assess dietary isoflavone and lignan intake. Cognitive functions were measured in three aspects: memory, processing capacity and speed, and executive function. The data revealed that cognitive function was not linked with dietary isoflavone intake. In contrast, high lignan intake was associated with better processing capacity and speed, as well as executive function [50]. In comparison to the above reports, the HAAS found opposite effects of soya on cognitive function. In this cross-sectional trial, volunteers were chosen from two different rural sites (n = 719, 52–98 years). Analyses were controlled for age, sex, education, site, and intake of other foods. Cognitive function was quantified using a word learning test sensitive to dementia and soy consumption was assessed using Food Frequency Questionnaire items. The results showed that high tofu consumption was associated with worse memory, whereas high tempe consumption was independently related to better memory, particularly in participants >68 years of age. It is unknown whether these negative results could be attributed to phytoestrogen levels or to its potential toxins. A possible explanation for this could be that tempe contains high levels of phytoestrogens, but due to fermentation also exhibits high folate levels, which may exert protective effects [147].

In a double blind trial involving healthy postmenopausal women (n = 350/45–92 years), the effect of daily isoflavones in the diet vs. dosing was tested on cognitive functions. In this trial, subjects were randomly dosed with 25 mg of isoflavone-rich soy protein (91 mg of aglycone weight of isoflavones: 52 mg of genistein, 36 mg of daidzein, and 3 mg glycitein) (structure of genistein, daidzein and glycitein, Figure 4B–D) or a milk protein-matched placebo. The endpoint for quantitating cognitive function was a composite of the weighted sum of 14 neuropsychological test score changes. The outcome of the trial disclosed that there was no significant between-group difference on the change from baseline for global cognition. Greater improvement was seen in secondary analyses on a visual memory factor in the isoflavone group. No significant difference was observed in the between-group differences on three other cognitive factors or individual test scores, and no significant difference within a subgroup of younger postmenopausal women was observed. Thus, long-term dietary soy isoflavone supplementation in traditional Asian diets has no effect on global cognition in healthy postmenopausal women but may improve visual memory [148]. Research suggests that higher dietary or supplemental intake of isoflavones and lignans is related to better cognitive performance in middle-aged and older women. Considering these observations, a longitudinal analysis of dietary phytoestrogens and cognitive performance in African-American, white, Chinese, and Japanese women undergoing the menopausal transition was conducted. The East Boston Memory, Digits Span Backward, and Symbol Digit Modalities were included as test parameters, and the Food Frequency Questionnaire was used to assess phytoestrogen intake. The results showed that coumestrol and isoflavone intake was 10 and 25 times less, respectively, in non-Asian than that in Asian participants. Asian women with high isoflavone intake performed better on processing speed during late perimenopause and postmenopause, but high-isoflavone Asian consumers performed worse on verbal memory during early perimenopause and postmenopause. Thus, the benefit of high lignan and not coumestrol consumption on verbal memory was only seen during perimenopause. It seems that the cognitive benefits of dietary phytoestrogens are small and are class specific, vary by cognitive domain and menopause stage, and differ among ethnic groups [149]. 

### 2.10. Triterpenoid Saponins from Polygala tenuifolia

*Polygala tenuifolia* (family Polygalaceae) is a reputable herb used in TCM for its memory enhancing effects. Radix Polygalae (RP), the root of *P. tenuifolia* is used in more than half of the most famous Chinese prescriptions to increase intelligence [150]. Polygalasaponins are the main constituents responsible for cognitive improvement by this plant [151,152,153]. There is a growing support for improved memory function in many animal models [151,152,153]. BT-11, an ethanolic extract from the *Polygala* root is effective against scopolamine-induced cognitive impairment during the passive avoidance response and water maze tests [154] as well as stress-induced [155] memory deficits in rats. Most saponins in RP are derivatives of presenegenin (Figure 4E).

The hydrolysate of polygalasaponins at doses of 50 and 100 mg/kg ameliorates amyloid β-protein (Aβ)_25*−*35_-induced amnesia probably via its antioxidant properties [156]. AChE activity is inhibited *in vitro* by an 80% ethanol extract of *P. tenuifolia* [151,157]. Tenuifolin is mostly a mixture of saponins [158] extracted from RP. Onjisaponin F, a constituent in *P. tenuifolia*, increases choline acetyltransferase mRNA level in rat basal forebrain cells, and oral administration of tenuifolin extracted from RP at doses of 0.02, 0.04, and 0.08 g/kg/day for 15 days evidently improves latency and the number of errors in aged mice. Moreover, tenuifolin at the same doses increases the relative levels of norepinephrine and dopamine in the hippocampus of aged mice and decreases AChE activity in the cortex [153]. A root extract of *P. tenuifolia* at 2 mg/kg/ day for 14 days also induces proliferation of a stem cell population in the rat hippocampal CA1 region [159]. Another study investigated the cognitive enhancing effect of polygalasaponin XXXII (PGS32), a triterpenoid saponin isolated from the roots of *P. tenuifolia.* PGS32 was administered at 0.125, 0.5, or 2 mg/kg and showed cognition enhancing effects in a mice scopolamine model possibly through improvements in synaptic transmission, activation of the mitogen-activated protein kinase cascade and enhancement of brain-derived neurotropic factor levels [160]. 

In a randomized, double-blind, placebo-controlled comparison study BT-11 was evaluated in elderly humans for memory enhancing effects. The authors used the Consortium to Establish a Registry for AD Assessment Packet (CERAD) and the MMSE for evaluating the memory enhancing effect. CERAD scores increased more significantly in the BT-11-treated group (n = 28) than those in the placebo-treated group (n = 25). Moreover, mean scores on word list recognition, constructional recall and praxis, and the Modified Boston Naming Test improved markedly in the BT-11-treated group compared to those in the placebo-treated group. BT-11 was effective for enhancing cognition in elderly humans and might be useful as a nutraceutical for its memory improving effects [29]. Another study from the same group was performed in healthy human volunteers on the memory enhancing property of BT-11. The participants were given capsules of BT-11 or placebo three times daily for 4 weeks. The Korean version of the California Verbal Learning Test (K-CVLT) and the Self-Ordered Pointing Test (SOPT) were used to assess verbal memory and working memory, respectively. A significant increase was observed in immediate recall in the BT-11-treated group compared to that in the placebo, as assessed by K-CVLT. Moreover, BT-11 significantly reduced the number of errors on the SOPT as compared with placebo treatment [30].

## 3. Potential Promising Compounds

*Acorus gramineus* and *Acorus calamus Linn.* are plants in the family Acoraceae. The main active compounds in these plants, α-asarone and β-asarone, are the most promising candidates for eliciting cognitive improvement. *A. gramineus* is listed in the Korean pharmacopeia for several pharmacological activities and has been used for hundreds of years for its beneficial effects on learning and memory and an antiaging effect in India and China [161]. α-Asarone and β-asarone have been reported for their AchE inhibition effects *in vitro* [162]. Several studies have shown cognitive enhancing effects of this plant. In our lab, α-asarone showed reversal of scopolamine-induced amnesia and inhibited AChE activity *in vivo* [163]. 

*Curcuma longa* (turmeric) is a perennial herb in the Zingiberaceae family. The main active constituent found in the rhizomes of turmeric is curcumin (diferuloylmethane), a polyphenol used extensively in Ayurveda [164]. Curcumin has demonstrated efficacy in several models of learning and memory impairment, including protective effects against phenytoin-induced cognitive impairment [165], scopolamine-induced amnesia [166], cigarette smoke-induced cognitive impairment [167], arsenic-induced cholinergic dysfunction [168], and intracerebral streptozocin-induced memory impairment [169] and it reverses impaired cognition and neuronal plasticity induced by chronic stress [170]. Focus has shifted to the neuroprotective effects of curcumin on cognition, as initial data have shown that regular curcumin intake may be related to better cognitive function in the elderly [171]. 

*Crocus sativus* L. commonly known as saffron, belongs to the Iridaceae family. The main chemical constituents are water-soluble carotenoids (crocins) small amounts of monoterpene aldehydes and its glucosides (safranal and picrocrocin), and flavonoids (quercetin and kaempferol) [172], which significantly antagonize scopolamine-induced amnesia [173]. Saffron from *Crocus sativus* was safe and effective in a randomized, placebo controlled, double-blind study in patients with mild to moderate AD for 16 [174] and 22 weeks [175]. 

*Melissa officinalis* (lemon balm) is a cultivated perennial lemon scented herb used in TCM as a tea for its nerve calming and spasmolytic effects [176]. This plant is used by the food industry to flavor different products due to its particular taste. *M. officinalis* modulates mood and cognitive performance following acute administration in a randomized, placebo-controlled, double-blind, balanced-crossover study [177], ameliorates agitation and improves quality of life in patients suffering from severe dementia [178], has value managing mild to moderate AD, and has a positive effect on agitation in such patients [179].

Moreover, consumption of coffee is also considered beneficial for learning and memory. A study showed that caffeine consumption restores memory performance and abrogates diabetes-induced loss of nerve terminals and astrogliosis in an animal model [180]. Ingestion of caffeine has no effect on initial mood or working memory, but it does improve encoding of new information and counteracts fatigue that developed over a test session in humans [181]. Furthermore, a diet rich in flavonols is associated with lower rates of dementia [182]. Flavonoid-rich food or beverages such as tea, cocoa, and blueberry juice have positive influences on learning and memory [42,183,184].

## 4. Conclusions

The use of herbal products has increased tremendously in Western societies and developing countries. Herbal medicines are perceived as natural, gentle, and safe treatments compared to those of synthetic drugs. Numerous natural products have been used traditionally as memory enhancers and provide promising effects to improve quality of life in terms of improving cognitive function associated with senescence. Contrary to traditional belief, some medicines do not fit and have failed to perform in the established test systems. This article has reviewed some of the plants and their active constituents that have been used in traditional medicine, including Ayurveda, Chinese, European and other Asian countries, for their clinical cognitive-enhancing and antidementia effects. Depending upon the mechanism of action and interference with learning and memory circuits, therapeutic intervention can either show symptomatic effect or even halt disease progression, *i.e.*, show disease modifying effect. AChE inhibitors (galantamine and HupA) provide disease-modifying therapies for AD. Naturally active substances usually are good lead molecules, but unlikely to meet the demands for druggability. Therefore, it is necessary to modify and optimize these structural phenotypes. The strategy of structural modification will help us to increase potency, selectivity, improve physico-chemical, biochemical and pharmacokinetic properties and eliminate or reduce side effects.

Most of the research reporting delay in the progression of AD is based on inhibiting the activity of AChE. Compounds such as galantamine and HupA act on AChE and are being prescribed for patients with AD. In the traditional practice of medicine, some of these plant products are already used as dietary supplements in developed countries for their memory enhancing effects. Still many traditional plants with the ability to improve memory function remain to be tested in clinical trials, which might provide therapeutic benefits in patients with AD or increase the quality of life in elderly people by improving their cognitive functions. A strong relationship exists between the traditional use of herbal plants and their mechanism of action, as identified in the current scenario using several pharmacological approaches. Several active compounds including glantamine and HupA have been isolated from these plants and their mechanism of action on learning and memory is well characterized. There is need to perform more studies with differences in study designs, number of subjects, different fractions of the compound used, doses, differences in sensitivities or neurocognitive tests, and specific patient with memory impairment for development of these herbal compounds as drug candidate in near future. 
